# Metabolic activity of hydro-carbon-oxo-borate on a multispecies subgingival periodontal biofilm: a short communication

**DOI:** 10.1007/s00784-021-03900-0

**Published:** 2021-03-28

**Authors:** Jamil Awad Shibli, Thayane Furtado Rocha, Fernanda Coelho, Ticiana Sidorenko de Oliveira Capote, Sybele Saska, Marcelo A. Melo, João Marcos Spessoto Pingueiro, Marcelo de Faveri, Bruno Bueno-Silva

**Affiliations:** 1 Department of Periodontology and Oral Implantology, Dental Research Division, Guarulhos University, Praça Tereza Cristina, 88, Centro, Guarulhos, SP 07023-070 Brazil; 2 Department of Morphology, São Paulo State University (UNESP), School of Dentistry, Araraquara, São Paulo Brazil; 3 M3 Health Industria e Comercio de Produtos Medicos, Odontologicos e Correlatos S.A., Jundiaí, SP Brazil

**Keywords:** Metaboloci activity, Hydro-carbon-oxo-borate, Periodontal biofilm, Peri-implantitis, Periodontitis, Periodontal pathogens

## Abstract

**Objective:**

This study evaluated the metabolic activity of hydro-carbon-oxo-borate complex (HCOBc) on a multispecies subgingival biofilm as well as its effects on cytotoxicity.

**Materials and methods:**

The subgingival biofilm with 32 species related to periodontitis was formed in the Calgary Biofilm Device (CBD) for 7 days. Two different therapeutic schemes were adopted: (1) treatment with HCOBc, 0.12% chlorhexidine (CHX), and negative control group (without treatment) from day 3 until day 6, two times a day for 1 min each time, totaling 8 treatments and (2) a 24-h treatment on a biofilm grown for 6 days. After 7 days of formation, biofilm metabolic activity was determined by colorimetry assay, and bacterial counts and proportions of complexes were determined by DNA-DNA hybridization. Both substances’ cytotoxicity was evaluated by cell viability (XTT assay) and clonogenic survival assay on ovary epithelial CHO-K1 cells and an osteoblast precursor from calvaria MC3T3-E1 cells.

**Results:**

The first treatment scheme resulted in a significant reduction in biofilm’s metabolic activity by means of 77% by HCOBc and CHX treatments versus negative control. The total count of 11 and 25 species were decreased by treatment with hydro-carbon-oxo-borate complex and CHX, respectively, compared with the group without treatment (*p* < 0.05), highlighting a reduction in the levels of *Porphyromonas gingivalis*, *Tannerella forsythia*, *Prevotella intermedia*, and *Fusobacterium periodontium*. CHX significantly reduced the count of 10 microorganisms compared to the group treated with HCOBc (*p* < 0.05). HCOBc and CHX significantly decreased the pathogenic red-complex proportion compared with control-treated biofilm, and HCOBc had even a more significant effect on the red complex than CHX had (*p* ≤ 0.05). For the second treatment scheme, HCOBc complex and CHX significantly decreased 61 and 72% of control biofilms’ metabolic activity and the counts of 27 and 26 species, respectively. HCOBc complex did not significantly affect the proportions of formed biofilms, while CHX significantly reduced red, orange, and yellow complexes. Both substances exhibited similar cytotoxicity results.

**Conclusions:**

This short communication suggested that the HCOBc complex reduced a smaller number of bacterial species when compared to chlorhexidine during subgingival biofilm formation, but it was better than chlorhexidine in reducing red-complex bacterial proportions. Although HCOBc reduced the mature 6-day-old subgingival multispecies biofilms, it did not modify bacterial complexes’ ratios as chlorhexidine did on the biofilms mentioned above. Future in vivo studies are needed to validate these results.

**Clinical relevance:**

HCOBc complex could be used to reduce red-complex periodontal bacterial proportions.

## Introduction

There has been an ongoing search for antimicrobial agents to use as an adjuvant to treat periodontal diseases, and/or peri-implantitis or mucositis is relatively constant over the literature. Several agents have been used in dentifrices, mouth rinses, and gels to control and/or prevent these diseases [1, 2]. The most common antimicrobial products in daily use are chlorhexidine (CHX), cetylpyridinium, triclosan, and several natural products such as aloe vera propolis [3–7].

Recently, a product based on hydro-carbon-oxo-borate complex (HCOBc) was made available, increasing the oxygen level in tissues, thus leading to improved tissue healing. The product is formed by chemical complexation of peroxoborate (NaBO_3_._n_H_2_O) with specific vehicles such as glycerol and cellulose sodium perborate-1,2-diol-glycerol/cellulose-ester adducts (hydro-carbon-oxo-borate complex). These complexes have the benefit of being capable of acting more specifically on target sides. Because of the nature of these complexes, there will be less tendency to form molecular oxygen when the complex decomposes when compared with the decomposition of hydrogen peroxide. Properties of reactive oxygen species (ROS) in periodontal disease have previously been demonstrated in the literature. It is known that immune cells can produce ROS to exert antibacterial activities. At the same time, excessive ROS production is also cytotoxic and may contribute to tissue destruction in periodontal/peri-implant disease [8, 9].

Few studies have evaluated the oxygenating products. In vitro and in vivo, previous studies demonstrated antibacterial activity and similar effects of mouthwash composed by oxygenating agent (Ardox-X®) compared to fluoride and chlorhexidine mouthwashes on the microbiota of healthy subjects [10, 11]. Specifically to the same products evaluated here, there was only one study comparing antiplaque and antigingivitis efficacy of HCOBc with triclosan-containing toothpastes [12]. The scarcity of up-to-date data on the use of this strategy has not prevented the increasing use of these products in dentistry. Therefore, the present study aimed to assess hydro-carbon-oxo-borate complex gel’s metabolic activity on the in vitro model of a subgingival multispecies biofilm in comparison with chlorhexidine.

## Material and methods

### Biofilm formation

The following species were used to form multispecies biofilm: *Actinomyces naeslundii* ATCC 12104, *Actinomyces oris* ATCC 43146, *Actinomyces gerencseriae* ATCC 23840, *Actinomyces israelii* ATCC 12102, *Actinomyces odontolyticus* ATCC 17929, *Streptococcus sanguinis* ATCC 10556, *Streptococcus oralis* ATCC 35037, *Streptococcus intermedius* ATCC 27335, *Streptococcus gordonii* ATCC 10558, *Streptococcus mitis* ATCC 49456, *Aggregatibacter actinomycetemcomitans* ATCC 29523, *Capnocytophaga ochracea* ATCC 33596, *Capnocytophaga gingivalis* ATCC 33624, *Eikenella corrodens* ATCC 23834, *Capnocytophaga sputigena* ATCC 33612, *Streptococcus constellatus* ATCC 27823, *Eubacterium nodatum* ATCC 33099, *Fusobacterium nucleatum vincentii* ATCC 49256, *Parvimonas micra* ATCC 33270, *Fusobacterium nucleatum polymorphum* ATCC 10953, *Campylobacter showae* ATCC 51146, *Fusobacterium periodonticum* ATCC 33693, *Prevotella intermedia* ATCC 25611, *Porphyromonas gingivalis* ATCC 33277, *Tannerella forsythia* ATCC 43037, *Eubacterium saburreum* ATCC 33271, *Streptococcus anginosus* ATCC 33397, *Selenomonas noxia* ATCC 43541, *Propionibacterium acnes* ATCC 11827, and *G. morbillorum* ATCC 27824.

The majority of the species were grown on tryptone soy agar with 5% sheep blood under anaerobic conditions (85% nitrogen, 10% carbon dioxide, and 5% hydrogen), except *Eubacterium saburreum* subsp. that was cultured on fastidious anaerobic agar with 5% sheep blood. *Porphyromonas gingivalis* was grown on tryptone soy agar with yeast extract enriched with 1% hemin, 5% menadione, and 5% sheep blood. For *T. forsythia* the media used contained tryptone soy agar with yeast extract enriched with 1% hemin, 5% menadione, 5% sheep blood, and 1% N-acetylmuramic acid. After 24 h of growth, all species were transferred to tubes with BHI culture medium (Becton Dickinson, Sparks, MD) supplemented with 1% hemin.

After 24 h, the optical density (OD) was adjusted to that inoculum would have approximately 10^8^ cells/ml of each species. The individual cell suspensions were diluted, and 100-μl aliquots containing 10^6^ cells of each species were mixed with 11,900 μl of BHI broth supplemented with 1% hemin and 5% sheep blood to give a final biofilm inoculum of 15 ml. Therefore, each species’ final inoculum was 1 × 10^4^ except for *P. gingivalis* to which 2 × 10^4^ were added.

The Calgary Biofilm Device, consisting of a 96-well plate (Nunc; Thermo Scientific, Roskilde, Denmark), was used to develop the multiple biofilm species models. An aliquot of 150-μl inoculum containing 10^4^ cells of each species was added per well, and was covered with a 96-pin lid for bacterial inoculations of 96-well plates (Nunc TSP system; Thermo Scientific, Roskilde, Denmark). The coated plates were incubated at 37 °C under anaerobic conditions. After 72 h of incubation, plate covers were transferred to fresh 96-well plates with fresh broth (BHI broth supplemented with 1% hemin and 5% sheep blood) and kept at 37 °C under anaerobic conditions for 7 days of biofilm formation [13, 14].

### Biofilm treatments

#### Effects on biofilm formation—twice-daily treatment scheme

Treatments began on 72-h biofilm, and were performed twice a day for the next 4 days. Biofilm-coated pins were transferred to 96-well plates containing 150 μl of hydro-carbon-oxo-borate complex (BlueM® gel, Curitiba, PR, Brazil), chlorhexidine 0.12% (CHX - Periogard, Colgate), and culture medium (for the non-treated biofilm-coated pins); each treatment lasted for 1 min, and then, biofilm-coated pins were returned to the original culture medium [15].

#### Effects on formed biofilm—24-h treatment scheme

Biofilms grew for 6 days, with media being changed on day 03. Then treatments with hydro-carbon-oxo-borate complex gel, chlorhexidine 0.12%, and culture medium (for the non-treated biofilm-coated pins) were performed 24 h. To perform treatments, 75 μl of agents were dispensed into the well with 75 μl of culture medium. After 24 h incubated under anaerobiosis, biofilms were collected to perform metabolic activity tests and checkerboard analysis [16].

### Biofilm metabolic activity

The percentage reduction in biofilm metabolic activity was determined using 2,3,5-triphenyltetrazolium chloride (TTC) (catalog no. 17779; Fluka analytical) and spectrophotometry. TTC is used for differentiation between metabolically active and inactive cells. The white substrate is enzymatically reduced to red formazan 1,3,5-triphenyl (TPHP) by live bacterial cells due to several dehydrogenases. The change in substrate color is read by spectrophotometry to determine the reduction rate, which is used as an indirect measure of bacterial metabolic activity. To measure biofilms’ metabolic activity, the pins were transferred to plates with 200 μl per well of fresh BHI medium containing 1% hemin with 10% of a 1% TTC solution. The plates were incubated at 37 °C under anaerobic conditions for 8 h. The TTC conversion was read at 485 nm using a spectrophotometer [14, 15].

### DNA-DNA hybridization (checkerboard DNA-DNA)

Three 7-day biofilm-coated pins of each group were transferred to Eppendorf tubes containing 100 μl of TE buffer (10 mM Tris-HCl, 1 mM EDTA [pH 7, 6]), and then 100 μl of 0.5 M NaOH were added. The tubes containing the pins and the final solution were boiled for 10 min. The solution was neutralized with the addition of 0.8 ml of ammonium 5 M. The samples were analyzed individually for the presence and quantity of the 30 bacterial species, using the DNA-DNA hybridization technique. Briefly, upon lysis of the samples, the DNA was plated onto a nylon membrane using a Minislot device (Immunetics, Cambridge, MA). After DNA attachment to the membrane, it was placed in a Miniblotter 45 (Immunetics). Digoxigenin labeled with DNA probes of the entire genome for the subgingival species used were hybridized to individual lanes of Miniblotter 45. After hybridization, the membranes were washed, and DNA probes were detected using a specific antibody to digoxigenin conjugated to phosphatase alkaline. The signals were detected using AttoPhos substrate (Amersham Life Sciences, Arlington Heights, IL), and the results were obtained using Typhoon Trio Plus (Molecular Dynamics, Sunnyvale, CA). Two lanes in each race contained the standards with 10^5^ and 10^6^ cells of each species. Signals obtained with the Typhoon Trio were converted to absolute counts by comparing the patterns on the same membrane. Failure to detect a signal was recorded as zero. The values obtained after hydro-carbon-oxo-borate complex and chlorhexidine treatments were compared with the values of negative controls. Counts below the method detection limit (1 × 10^4^) were considered zero to calculate individual bacterial species’ mean counts [17].

### Cell cultures

Chinese hamster ovary cells (CHO-K1) and osteoblast precursor cell line derived from *Mus musculus* calvaria (MC3T3-E1) were seeded in Ham-F10 + DMEM medium (1:1) (Sigma®, St. Louis, MO, USA), and α-MEM culture medium, respectively, supplemented with 10% FBS and kanamycin (1%), and incubated at 37 °C with 5% CO_2_ and used at third passage. Treatments were in duplicate and included negative controls. Three independent experiments were conducted for each assay.

For cytotoxicity tests, eluates from the hydro-carbon-oxo-borate complex were made according to the International Organization for Standardization (ISO 10993-12 Biological evaluation of medical devices—Part 12: Sample preparation and reference materials), considering the weight (0.1g/ml). The hydro-carbon-oxo-borate complex was diluted in 1:1Ham-F10 + D-MEM medium (Sigma®, St. Louis, MO) (for CHO-K1 cell culture), and in α-MEM (for MC3T3-E1 cell culture), without fetal bovine serum (FBS). CHX (0.1 μl/ml) were immersed in both culture media.

### Cytotoxicity tests

#### XTT assay (cell viability)

CHO-K1 (2 × 10^4^ cells) were seeded in 24-well plates in a culture medium (1 ml, HAM-F10:DMEM; 1:1). MC3T3-E1 cells (5 × 10^3^) were seeded in 48-well plates in a volume of 1 ml of α-MEM medium (1:1). Both cultures were supplemented with 10% of FBS at 37 °C, in 5% CO_2_. After 24 h, the cells were washed with phosphate-buffered saline (PBS) solution and then treated with the materials for 24 h. Each well was supplemented with 10% FBS. The negative control (NC) consisted of cells with a culture medium supplemented with 10% FBS without any treatment (untreated controls). For positive control (PC), the cells were treated with doxorubicin (3.0 μg·ml^−1^) for 24 h. After treatment, the cultures were washed with PBS solution, and immediately 500 μl of DMEM without phenol red were added, followed by 60 μl of the XTT/electron solution (Cell Proliferation Kit II—Roche Applied Science). After 3 h of reaction, the supernatant was transferred to a 96-well culture plate, and then the absorbance was measured using a microplate reader (VersaMax, Molecular Devices, Sunnyvale, CA) at 492 and 690 nm.

#### Clonogenic survival assay

CHO-K1 (5 × 10^4^ cells) were seeded in 24-well plates in culture medium (1 ml, HAM-F10:DMEM; 1:1); MC3T3-E1 cells (6 × 10^4^) were seeded in 48-well plates in a volume of 1 ml of α-MEM medium (1:1). Both cultures were supplemented with 10% of FBS at 37 °C, in 5% CO_2_. After 24 h, the cells were washed with PBS solution and then treated with the materials, supplemented with 10% FBS for 24 h. For PC, cells were treated with doxorubicin (0.3 μg·ml^−1^) for 4 h. After treatment, exponentially growing cells were seeded in the number of 150 cells per 25-cm^2^ culture flasks (for CHO-K1), and 300 cells per 25-cm^2^ culture flasks. The culture flasks were incubated at 37 °C, in 5% CO_2_, for 7 days without medium changes. The colonies were fixed with methanol:acetic acid:water (1:1:8 *v*/*v*/*v*) for 30 min and stained with Giemsa 1:20 for 20 min. The number of colonies counted in the NC was considered 100%. From this, survival fractions (SF) were obtained: SF = number of colonies counted in each treatment × 100/number of colonies observed in NC.

### Statistical analysis

Mean counts of each bacterial species were analyzed by Mann-Whitney *U* test. XTT assay data were analyzed by Kruskal-Wallis analysis followed by Dunn as post hoc. Metabolic activity of biofilm and clonogenic survival assay, ANOVA followed by the Tukey tests, were applied. The level of significance was 5%.

## Results

**Fig. 1:**
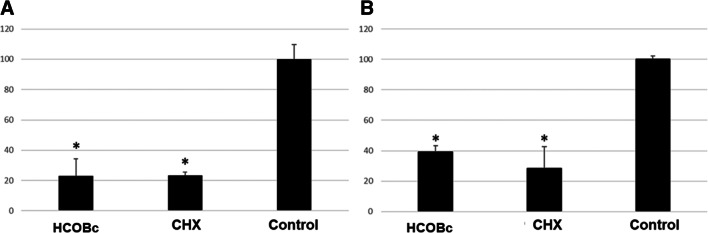
Results (mean and standard deviation) of metabolic activity of multispecies biofilms treated with hydro-carbon-oxo-borate complex gel (HCOBc), chlorhexidine 0.12% (CHX), and culture medium (control). Control biofilms were considered 100% metabolic activity. **a** Twice daily scheme and **b** 24-h treatment on formed biofilms. Asterisks indicate statistical significance by ANOVA followed by Tukey post hoc test for the control group (*p* ≤ 0.05)

HCOBc and chlorhexidine were able to significantly reduce 77% of biofilms’ metabolic activity in the twice-daily treatment scheme compared to the negative control culture medium treatment (*p* ≤ 0.05; Fig. 1a). In the analysis of formed biofilms within the 24-h treatment scheme, HCOBc and chlorhexidine significantly decreased 61 and 72% of negative control biofilms’ metabolic activity, respectively (*p* ≤ 0.05; Fig. 1b).

**Fig. 2:**
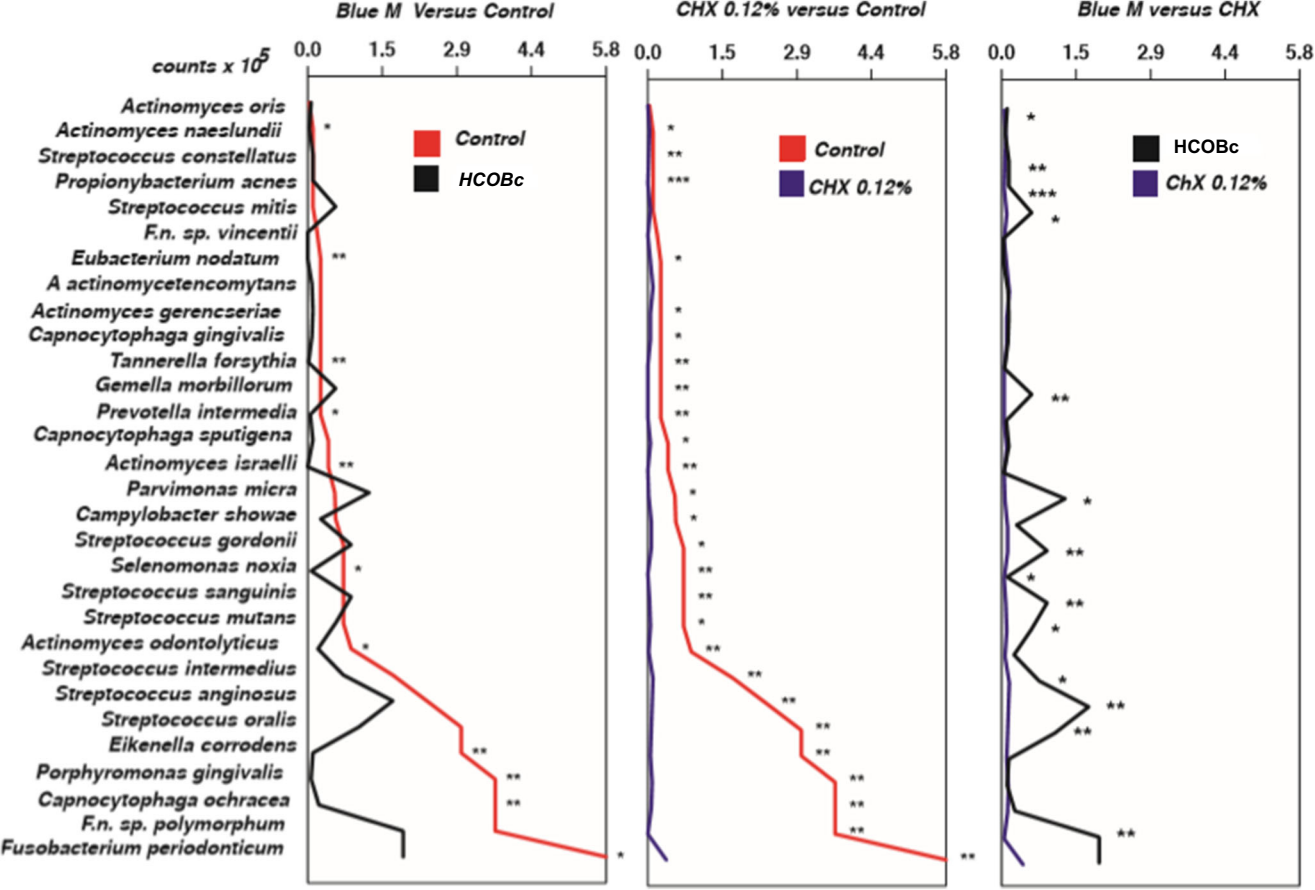
Mean bacterial count (×10^5^) of biofilms treated twice daily with hydro-carbon-oxo-borate gel (HCOBc), chlorhexidine 0.12% (CHX), and culture medium (control). Data were analyzed using the Mann-Whitney test (*p < 0.05, **p < 0.01, ***p < 0.001)

Figure 2 shows the counts of each bacterial species of biofilms treated by the twice-daily scheme. HCOBc significantly reduced counts of 11 out of 32 species compared with the negative control (*A. naeslundii*, *E. nodatum*, *P. intermedia*, *A. israelli*, *T. forsythia*, *S. noxia*, *A. odontolyticus*, *E. corrodens*, *P. gingivalis*, *C. ochracea*, *F. nucleatum polymorphum*) while chlorhexidine 0.12% significantly decreased counts of 26 out 32 species when compared with the negative control group, including *A. naeslundii*, *S. constellatus*, *P. acnes*, *E. nodatum*, *A. gerencseriae*, *C. gingivalis*, *T. forsythia*, *G. morbillorum*, *P. intermedia*, *C. sputigena*, *A. israelli*, *P. micra*, *C. showae*, *S. gordonii*, *S. noxia*, *S. sanguinis*, *S. mutans*, *A. odontolyticus*, *S. intermedius*, *S. anginosus*, *S. oralis*, *E. corrodens*, *P. gingivalis*, *C. ochracea*, *F. nucleatum polymorphum*, and *F. periodonticum*. In the comparison between the two antimicrobial agents, chlorhexidine reduced the counts of 11 species.

HCOBc and chlorhexidine significantly decreased the proportion of the disease associated red complex compared with the control-treated biofilm, and HCOBc had even a more significant effect on the red complex than chlorhexidine had (*p* ≤ 0.05). However, HCOBc also significantly reduced proportions of green and purple complexes regarding beneficial microbiota compared with the control and chlorhexidine groups (*p* ≤ 0.05). Moreover, hydro-carbon-oxo-borate increased the ratio of the complex called as others when compared only with the negative control group (*p* ≤ 0.05).

**Fig. 3:**
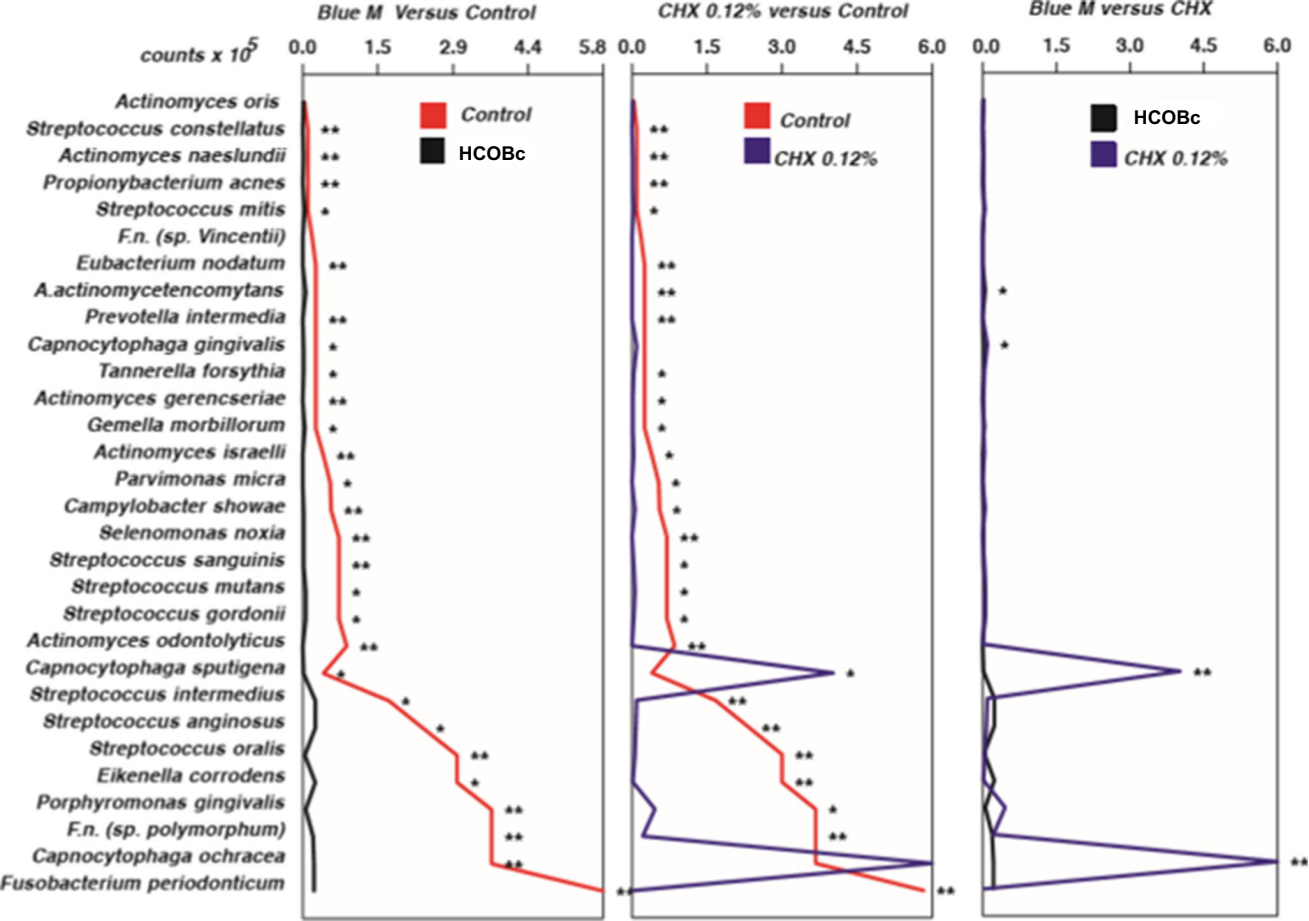
Mean bacterial count (×10^5^) of 6-day biofilms treated during the next 24 h with hydro-carbon-oxo-borate gel, chlorhexidine 0.12% (CHX), and culture medium (control). Data were analyzed using the Mann-Whitney test (*p < 0.05, **p < 0.01, ***p < 0.001)

Figure 3 demonstrates the results of 6-day formed biofilms treated by 24 h. HCOBc gel significantly reduced counts of 28 species (except *A. oris*, *F. nucleatum vicentii*, and *A. actinomycetemcomitans*) while chlorhexidine treatment significantly reduced the counts of 26 (except *A. oris*, *F. nucleatum vicentii*, and *C. gingivalis*) and increased counts of *C. sputigena.* In comparing the two antimicrobial agents, HCOBc significantly reduced counts of *C. gingivalis*, *C. ochracea*, and *C. sputigena* while CHX significantly reduced counts of *A. actinomycetemcomitans*.

HCOBc had no significant effect on the proportions of formed biofilms, while the positive control chlorhexidine significantly reduced proportions of pathogenic red and orange complexes and the beneficial yellow complex. Furthermore, CHX increased the beneficial green complex compared with the control group (*p* < 0.05).

**Fig. 4:**
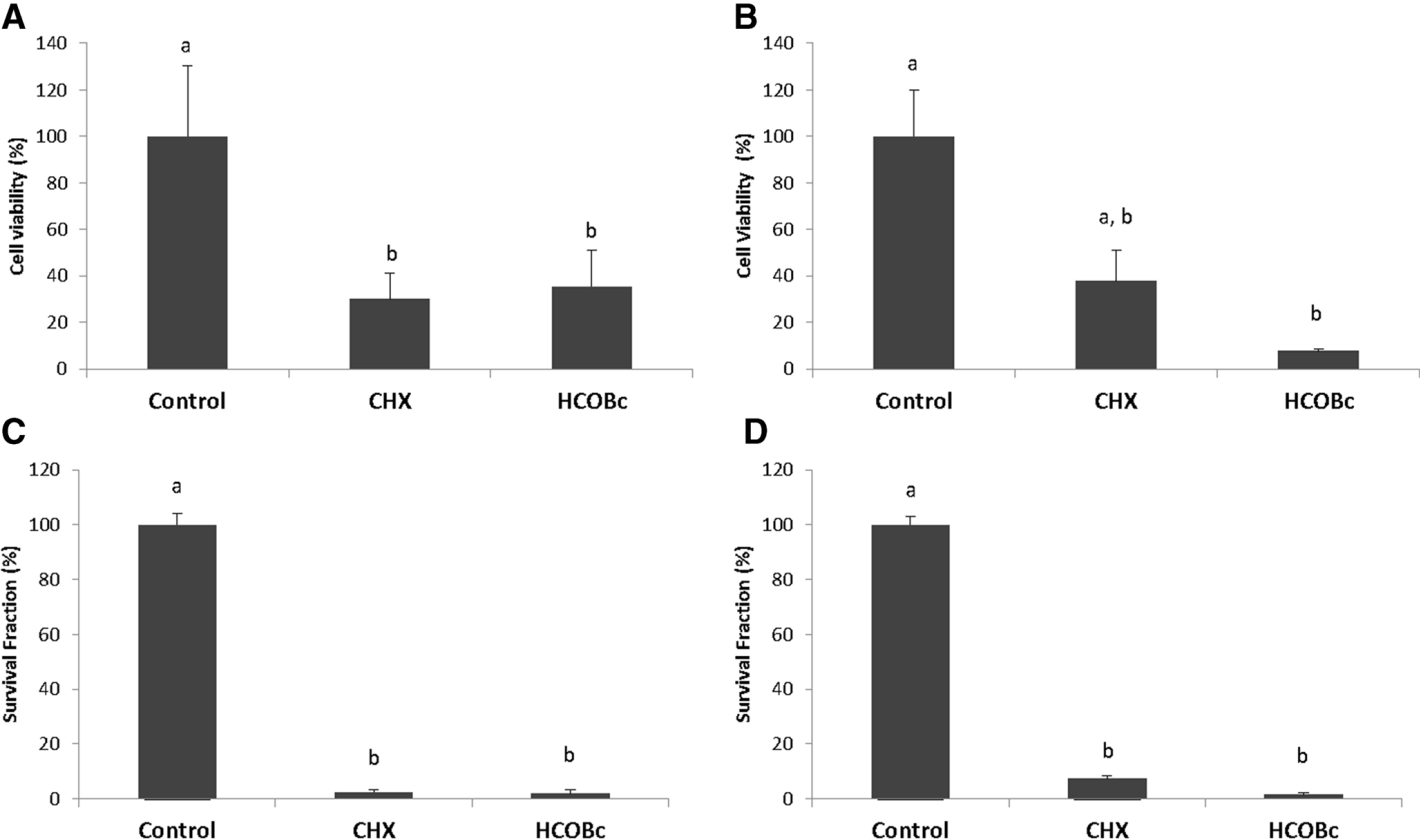
Cytotoxicity assessed by the XTT method (cell viability) on **a** CHO-K1 cells and **b** MC3TE-E1 and assessed by clonogenic survival assay. **c** CHO-K1 cells and **d** MC3TE-E1. Columns indicate mean cell viability or mean survival fraction (%). Bars indicate standard errors. Different letters mean statistical significance by Kruskal-Wallis followed by Dunn as post hoc test (*p* < 0.05)

Chlorhexidine 0.12% and hydro-carbon-oxo-borate had similar cytotoxicity effects by significantly decreasing the viability of CHO-K1 cells (*p* ≤ 0.05) (an epithelial cell line derived from the ovary of a Chinese hamster) by 70 and 65%, respectively (Fig. 4a). Hydro-carbon-oxo-borate significantly affected MEC3T3-E1 cells when compared with the control group (*p* ≤ 0.05). At the same time, CHX treatment did not show a significant difference when compared with both control and hydro-carbon-oxo-borate treatments (*p* ≥ 0.05; Fig. 4b).

Figure 4c and d depict, respectively, the results of the clonogenic survival assays on CHO-K1 cells and MC3TE-E1 cells revealed a decrease of cell proliferative capacity for both chlorhexidine and hydro-carbon-oxo-borate treatments in comparison with the non-treated cells (*p* < 0.05).

## Discussion

The effects of hydro-carbon-oxo-borate gel on subgingival biofilm formation and mature biofilm (6-day biofilm) were evaluated using a complex in vitro biofilm model. To evaluate antimicrobial effects on biofilm formation, treatments started on day 03 and were performed for 1 min each, two times per day, until day 06. In total, eight 1-min treatments were performed during the formation of 7-day biofilms. Hydro-carbon-oxo-borate reduced counts of 11 species, while the positive control chlorhexidine reduced counts of 26 species during biofilm formation compared with the negative treatment control group. Among bacteria affected by the hydro-carbon-oxo-borate reduction of red complex members, *P. gingivalis* and *T. forsythia*, and orange complex members, *P. intermedia* and *E. nodatum*, must be pointed out, since these bacteria are well-recognized periodontal pathogens detected in both periodontal and peri-implant diseases [18, 19]. These data also agree with previous study [10] that showed the inhibition of some Gram-negative bacteria such as *Veillonella*, *Tannarella*, *Campylobacter*, *Fusobacterium*, and *P. gingivalis.*

Hydro-carbon-oxo-borate had an excellent antibiofilm effect compared with biofilm treated with culture medium (negative control); however, compared with chlorhexidine, the scenario seems to be different. CHX reduced the counts of 11 species, highlighting *P. intermedia*, *S. gordonii*, and *S. mutans*, compared with hydro-carbon-oxo-borate-treated biofilms. *Prevotella intermedia* is a recognized orange complex pathogen that is a usual target in clinical trials concerning periodontal/dental implant diseases [20–22]. *Streptococcus gordonii* was recently considered a contributor to the establishment of the keystone pathogen *P. gingivalis* in the subgingival biofilm [23]. Lastly, an increase in sugar intake by the host favors modification of the subgingival biofilm by *S. mutans* to having a pathogenic microbiota profile rather than to a healthy state [24]; however, this dysbiosis has not yet been completely elucidated.

Recently, a novel antimicrobial approach was proposed, in which the elimination of disease-associated microorganisms was considered more relevant than eliminating the complete oral biofilm [25, 26]. This has made it necessary to analyze the proportions of beneficial and pathogenic microbiota. Hydro-carbon-oxo-borate had a remarkable effect on reducing the red complex, shown to be even better than chlorhexidine. In comparison, hydro-carbon-oxo-borate reduced green and purple health-associated complexes, an effect that was not observed in chlorhexidine-treated biofilm. Furthermore, hydro-carbon-oxo-borate increased the other complex’s proportion to which *S. mutans* belongs, ratifying earlier study [11]. This finding may raise a warning flag since *S. mutans* is known for their pathogenic action in caries disease [27].

Nevertheless, at this point, it is necessary to consider that this study has shown in vitro data. So far, hydro-carbon-oxo-borate exhibited positive (red complex reduction and yellow complex increase) and negative (purple and green complex reduction) effects, leading to the question: which will prevail after clinical use? Future in vivo studies should answer this question. Moreover, a recent article demonstrated similar effects on supragingival biofilm control between active oxygen and lactoferrin-containing toothpastes, and triclosan-containing toothpastes [12]. However, the report provided no clinical periodontal data and no analysis of subgingival biofilm.

Hydro-carbon-oxo-borate effects on a mature 6-day biofilm were also evaluated. In this scenario, hydro-carbon-oxo-borate’s results seemed similar to those of chlorhexidine since both antimicrobial agents reduced counts of 27 and 26 species, respectively (Fig. 3). However, here the challenge is how to keep the antimicrobial agent in contact with biofilm for 24 h in vivo. For this task, hydro-carbon-oxo-borate was manipulated as a gel, which is expected to have a better substantivity that needs to be analyzed in the future. Clinical studies seem to be an excellent strategy for this purpose.

Although hydro-carbon-oxo-borate reduced the number of total biofilm counts, the analysis of complex proportions revealed that hydro-carbon-oxo-borate had no significant effect on proportions of 6-day formed biofilm, even if the agent was retained in biofilm for 24 h. Although the hydro-carbon-oxo-borate reduced proportion of red complex from 15% (from negatively treated biofilms) to 10% and more than a half of orange complex (from 29% to 14%), these differences were not statistically significant. In this scenario, chlorhexidine reduced red and orange pathogenic complexes and increased the beneficial green complex. The high percentage of green complex in chlorhexidine-treated biofilms was due to high counts of *C. sputigena* and *C. ochracea* determined, as shown in Fig. 3.

Chlorhexidine is an antimicrobial agent frequently added to oral topical formulations and/or incorporated into local delivery systems such as gels. This agent has been studied for over four decades, and it is considered the gold standard to control oral plaque [5, 28, 29]. There is a discussion regarding whether chlorhexidine still deserves the title of the gold standard or not [30]. Despite the strong existing evidence that the use of a chlorhexidine mouthwash for 4–6 weeks or 6 months improves the mechanical hygiene procedures and reduces plaque accumulation, several studies have demonstrated that it has side effects such as tooth staining, causing taste disturbance and calculus formation after prolonged use for 4 weeks or longer [28]. Moreover, treatment of wounds with chlorhexidine is restricted due to its cytotoxicity towards human fibroblasts [31].

This in vitro study presents some limitations. The hydro-carbon-oxo-borate gel did not differ from chlorhexidine (Fig. 4) regarding cytotoxicity to CHO-K1 cells, an epithelial cell line derived from the ovary of the Chinese hamster, and on MEC3T3-E1, an osteoblast precursor cell line derived from *Mus musculus* (mouse) calvaria. These cells are commonly used for cytotoxic tests, and chlorhexidine is toxic to these cells [32–34]. Future studies need to address the mechanisms involved in its cytotoxic effects. Probably, other components present in the pharmaceutical formulation of the products are responsible for these types of results; however, future studies need to demonstrate this hypothesis.

The present in vitro study did not evaluate saliva’s impact, e.g., saliva-coating, on the polymicrobial colonization and co-aggregation. Saliva components are essential to mediate microbial attachment to oral surfaces and dental implants and restorative materials. The planktonic microbial surfaces interact with the saliva and facilitate the agglutination and eliminate the bacteria from the oral cavity [35].

Another limitation of our study lies in the different pharmaceutical formulations used for hydro-carbon-oxo-borate and chlorhexidine. While the first was a gel, the second was a mouth rinse. We decided to test the gel formulation due to several informal reports of dental clinicians attesting this product’s efficacy when used during peri-implantitis treatment. In addition, the majority of clinicians use the gel formulation, while chlorhexidine is widely used as mouthwash, as was done in this study.

It is crucial to bear in mind that the scientific evidence supporting the clinical treatment procedures is based on systematic reviews with meta-analysis and randomized clinical trials. Hence, the present study results should not support changes in the clinical treatment of periodontitis, but it may provide a basis for future in vivo studies.

In summary, hydro-carbon-oxo-borate lowered fewer bacterial species than chlorhexidine in reducing overall subgingival biofilm formation and was better than chlorhexidine in reducing red-complex bacterial proportions in an in vitro subgingival multispecies biofilm model. On the other hand, HCOBc also reduced the beneficial green and purple complexes compared to control-treated biofilms. Although hydro-carbon-oxo-borate reduced the total counts of 6-day subgingival multispecies biofilms, it did not change the complexes of these biofilms as chlorhexidine performed. Future in vivo studies should determine hydro-carbon-oxo-borate’s effectiveness as an adjuvant treatment for periodontal disease using in vivo studies.
